# Lipid-rich carcinoma of the breast: A report of two cases and a literature review

**DOI:** 10.3892/ol.2015.2924

**Published:** 2015-02-03

**Authors:** YIZI CONG, JUN LIN, GUANGDONG QIAO, HAIDONG ZOU, XINGMIAO WANG, XIAOHUI LI, YALUN LI, SHIGUANG ZHU

**Affiliations:** Department of Breast Surgery, Yantai Yuhuangding Hospital Affiliated to the Medical College of Qingdao University, Yantai, Shandong 264400, P.R. China

**Keywords:** breast, lipid-rich carcinoma, imaging, treatment, prognosis

## Abstract

Lipid-rich carcinoma of the breast is extremely rare with no standard guidelines for treatment with poor patient prognosis. In the present study, the clinical features, imaging results, pathology, immunohistochemistry, treatment and prognoses of two patients with lipid-rich carcinoma of the breast were analyzed. Two patients were admitted to the Yantai Yuhuangding Hospital Affiliated to the Medical College of Qingdao University (Yantai, Shandong, China) for examination of a palpable mass in the breast. Enlarged lymph nodes were found in the axilla of each patient. The results of mammography and echography imaging suggested the presence of malignancy. A modified radical mastectomy was performed in each patient, and pathological examination revealed atypical large vacuolated cells arranged in clusters and confirmed lipid-rich carcinoma and lymph node metastases. The tumor tissue of patient one was immunohistochemically positive for estrogen receptor (ER), p53, p120 and E-cadherin, and negative for progesterone receptor (PR) and human epidermal growth factor receptor 2 (HER-2), with a Ki-67 labeling index of 50%. The tumor tissue of patient two was immunohistochemically positive for p53, and negative for ER, PR, HER-2 and cytokeratin 5/6, with a Ki-67 labeling index of 30%. Post-surgery, patient one was administered chemotherapy for six cycles, radiotherapy and endocrine therapy in the form of anastrozole. Patient two was administered three cycles of chemotherapy without radiotherapy. Subsequent to being followed up for 25 months and 13 months, respectively, there was no evidence of recurrence or distant metastasis in patient one or two, respectively.

## Introduction

As an extremely rare subtype of invasive ductal carcinoma, lipid-rich carcinoma of the breast represents <1% of all breast malignant tumors (1). Lipid-rich carcinoma was classified as a unique type of breast tumor in the 2003 World Health Organization classification (2). The definition of lipid-rich carcinoma is controversial as it is unclear as to the number of vacuolated cells required to be present in order to confirm the diagnosis and the origin of the lipids in the neoplastic cells remains unclear (3). The usual differential diagnosis of lipid-rich carcinoma includes vacuolated or clear cell breast tumors and mainly glycogen-rich, apocrine and secretary carcinomas (4). The tumor presents with an aggressive clinical course and a poor prognosis (5). There are currently no standard guidelines for the treatment of lipid-rich carcinoma, however, chemotherapy is considered to be the most effective method. Only sporadic case reports of lipid-rich carcinomas exist in the literature. The current study reports two cases of lipid-rich carcinoma and presents the clinicopathological features in order to further aid in the elucidation of its biological behavior and prognosis. Written informed consent was obtained from the families of the patients.

## Case report

### Patient one

**Figure 1:**
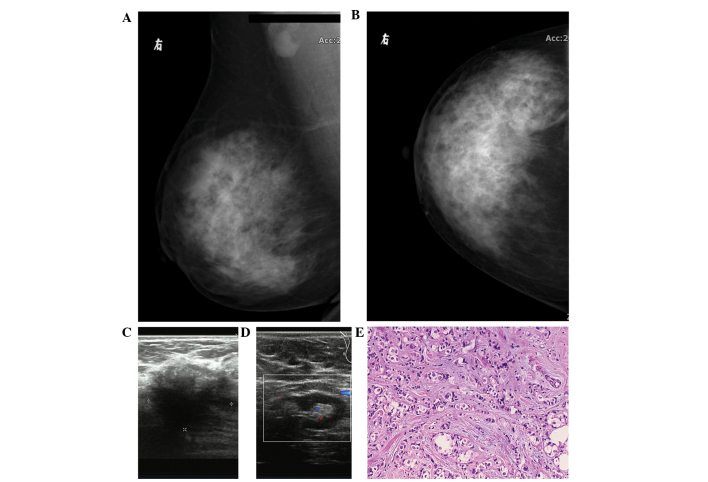
(A) Oblique and (B) axial mammography findings showing a twisted local structure with sand-like calcification in the superior external quadrant of the right breast. A significantly enlarged lymph node was observed in the right axilla. (C) Echography imaging showing a low echo 2.6×1.8-cm mass with an ill-defined margin. The mass was less regular in morphology, rough and presented with an uneven echo. Color Doppler flow imaging (CDFI) showing no blood flow signal detected in and around the mass. (D) Echography imaging showing a 2.6×1.2-cm hypoechoic nodule found in the right axilla, with a clear edge and a center of echo enhancement, and CDFI showing a small amount of blood flow in the nodule. (E) Lipid-rich carcinoma of the breast, with typical large, vacuolated cells arranged in clusters (hematoxylin and eosin staining; magnification, ×100).

A 55-year-old premenopausal female visited the Yantai Yuhuangding Hospital Affiliated to the Medical College of Qingdao University (Yantai, Shandong, China) in order to be examined for a palpable mass in the right breast, which had been found one week previously. A medical history was taken, and it was noted that the patient’s brother had previously succumbed to colon carcinoma. A physical examination of the patient revealed a 3.0×3.0-cm, non-tender mass in the superior external quadrant of the right breast. An enlarged lymph node (2.5×2.0 cm) was found in the right axilla. The node had not fused together and could move freely. No mass was palpable in the bilateral supraclavicular fossae. The tumor biomarkers of tumor-specific growth factor (TSGF), carcinoembryonic antigen (CEA), cancer antigen (CA)15-3, CA-125 and ferritin (FERR), and the routine hematological and biochemical parameters were within the normal ranges. Mammography revealed that the local structure of the gland was twisted with sand-like calcification and a significantly enlarged axillary lymph node (Fig. 1) and echography imaging revealed a mass with an ill-defined margin, which was less regular in morphology and an enlarged axillary lymph node (Fig. 1); these results suggested the presence of malignancy. The clinical diagnosis was of breast cancer. Following sufficient preparation, the patient received a modified radical mastectomy. Grossly, the tumor was gray-white, with an indistinct boundary measuring 5.5×2.0 cm. Microscopic examination revealed clustered atypical large vacuolated cells, which confirmed that the tumor was a lipid-rich carcinoma. The nipple and deep resection plane were not involved. Out of a total of 10 lymph nodes isolated, one exhibited tumor metastases. Immunohistochemical staining indicated that the carcinoma was positive for estrogen receptor (ER), p53, p120 and E-cadherin, and negative for progesterone receptor (PR) and human epidermal growth factor receptor 2 (HER-2). Immunostaining for D2-40 showed that the majority of lymphatics exhibited tumor emboli. In total, 50% of the tumor cells showed nuclear staining for Ki-67. Post-surgery, the patient was administered chemotherapy consisting of 80 mg/m^2^ intravenous (i.v.) epirubicin and 75 mg/m^2^ i.v. docetaxel on day 1, every three weeks for six cycles, radiotherapy (50 Gy) for five weeks and endocrine therapy consisting of 1 mg anastrozole once a day, for 20 months. Subsequent to being followed up for 25 months, the patient exhibited no signs of recurrence or distant metastasis.

### Patient two

**Figure 2:**
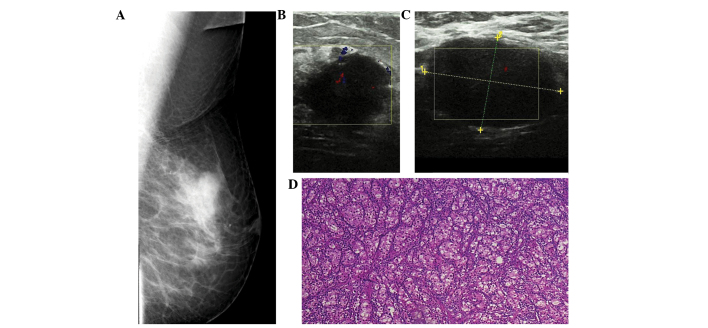
(A) Mammography findings of a 2.0×1.5-cm slightly high-density tumor with a local clear border in the superior external quadrant of the left breast. (B) Echography imaging showing a hypoechoic mass measuring 1.9×1.3 cm, with a less clear boundary, serrated edges, an uneven echo and rear echo attenuation. CDFI: a small amount of blood flow signal was detected in the mass. (C) Two hypoechoic nodules found in the right axilla; the larger measuring 3.0×2.0 cm with a clear boundary and uneven echo. Color Doppler flow imaging showing a small amount of blood flow in the nodules. (D) Lipid-rich carcinoma of the breast, with atypical large vacuolated cells arranged in clusters (hematoxylin and eosin staining; magnification, ×100).

A 56-year-old menopausal female was admitted to the Yantai Yuhuangding Hospital Affiliated to the Medical College of Qingdao University due to a painless lump in the left breast that had been found 10 days previously. The medical history showed that the patient’s sister also suffered from breast cancer. A physical examination revealed a 3.0×2.0-cm, non-tender mass in the upper outer quadrant of the left breast. An enlarged lymph node (3.0×2.0 cm) was found in the right axilla. The node had not fused together and could move freely. No mass was palpable in the opposite breast or in the bilateral supraclavicular fossae. The tumor biomarkers of TSGF, CEA, CA15-3, CA125 and FERR, and the routine hematological and biochemical parameters were within the normal ranges. The results of mammography and echography imaging (Fig. 2) supported the diagnosis of malignancy; mammography revealed a high density tumor with a local clear border and echography imaging revealed a mass with a less clear boundary, uneven echo and rear echo attenuation, in addition to enlarged lymph nodes in the right axilla. A left modified radical mastectomy was performed and a specimen was sent for histopathological analysis. Grossly, the tumor was hard with a gray cut surface and clear boundary measuring 2.2×1.5 cm. The pathological findings revealed that the tumor was lipid-rich carcinoma. The tumor cells were polygonal with abundant cytoplasm and eosinophilic granules. The nuclei were round and eosinophilic small nucleoli were visible. The nipple and deep resection plane were not involved. Of the 14 axillary lymph nodes sampled, the presence of metastasis was found in two. Upon immunohistochemistry analysis, the carcinoma was negative for ER, PR, HER-2 and CK5/6, and positive for p53. The Ki-67 labeling index was 30% in the tumor tissues. Post-surgery, the patient was administered three cycles of chemotherapy consisting of 80 mg/m^2^ i.v. epirubicin and 600 mg/m^2^ i.v. cyclophosphamide on day 1, every three weeks for one cycle, and 175 mg/m^2^ i.v. paclitaxel liposome on day 1, every three weeks for two cycles, without radiotherapy. Subsequent to being followed up for 13 months, local recurrence, positive axillary lymph nodes and distant metastasis were not found.

## Discussion

Lipid-rich carcinoma of the breast was first described in a study Aboumrad *et al* in 1963 (6), in which a carcinoma with lipid vacuoles in the neoplastic cells was reported and where the lesion was referred to as a lipid-secreting carcinoma. The term lipid-rich is now preferred, as it describes only the presence of the lipid substance in the cells, and not active lipid secretion, unless there is evidence of such in the neoplastic cells (4).

A review of the literature shows that the recorded patients with lipid-rich carcinoma ranged in age from 33 to 81 years old, and that only one case was exhibited in a male (2). In the majority of cases, the tumor presented as a breast mass or lump. The masses were all unilateral and equally distributed in the left and right breast, but were mostly localized in upper outer quadrant. As it is unclear as to the number of lipid-containing cells required to confirm the diagnosis of lipid-rich carcinoma, the definition of the tumor is not yet clearly established (3). Certain studies have suggested that >90% of the neoplastic cells should contain lipid droplets in lipid-rich carcinoma (2,7). The origin of the lipids in the neoplastic cells is also not clear. Normal epithelial breast cells can synthesize carbohydrates and proteins, but also lipids (4). Certain studies have hypothesized that malignant cells of lipid-rich carcinoma release lipid as a secretory product and not as a result of degeneration, as lipid vacuoles have been found in a cytoplasmic location close to an enlarged Golgi apparatus and huge endoplasmic reticulum, without the presence of autophagic vacuoles (8,9).

Analysis of the ultrastructure of lipid-rich carcinoma has been performed in a small number of cases, leading to differing results, however the presence of intracytoplasmic lipid droplets and globules isolated by distinct membranes and surrounded by a dense cytoplasmic rim was described in all cases (9).

As lipid-rich carcinoma is rare, the association between immunohistochemical subtypes and tumor aggressiveness has not been extensively studied. In the present study, each of the two cases was found to be HER-2 negative and one was identified as positive for hormonal receptors. In a previous study of 17 lipid-rich carcinomas, all cases were negative for steroid receptors, with the exception of one case, and all were HER-2-positive (1). In another study of 49 cases of the tumor, ER and/or PR positivity was found in 5 out of 49 (10.2%) cases, while 35 out of 49 (71.4%) cases were HER-2-positive (5). Machalekova *et al* (10) reported a single case of lipid-rich carcinoma and invasive ductal carcinoma in the same breast; this patient was negative for steroid receptors, and exhibited positive staining for HER-2 and p53, with a high Ki-67 proliferative index. Together, these data indicate that lipid-rich carcinomas usually exhibit HER-2 positivity, but are negative for hormonal receptors. The propensity of lipid-rich carcinomas for high levels of HER-2 expression may be responsible for the associated poor prognosis and short disease-free survival time (1,11). Ki-67 is also important with regard to the prognosis. In the present cases, an average of 40% of the tumor cells exhibited nuclear staining for Ki-67. Another study showed positive nuclear staining for Ki67 in >30% of tumor cells in 27 out of 49 (55.1%) patients, thereby displaying high proliferation activity (3). Tumor cells can also express prolactin receptor, indicating the use of prolactin in the genesis of this neoplasm (11).

The differential diagnosis of lipid-rich carcinoma includes vacuolated or clear cell breast tumors of primary and secondary origin, and mainly glycogen-rich, apocrine and secretary carcinomas, all of which possess a different metabolic product in their mainly foamy cytoplasm. Other potential tumors are clear cell myoepithelioma and myoepithelial carcinoma, clear cell sarcoma and metastatic renal cell carcinoma of clear cell type (5). One study has reported the positivity of S-100 protein as a potentially useful marker in the diagnosis of lipid-rich carcinoma (12).

No standard guidelines exist for the treatment of lipid-rich carcinoma. Radical surgery and systemic treatments are frequently used, but the benefit of these therapeutic approaches has not been demonstrated. Axillary lymph node dissection is usually essential due to the high rate of axillary lymph node metastasis. Lipid-rich carcinoma can develop metastases soon after surgery, and the most common metastatic sites include the lungs, liver and bones, therefore, systematic therapy plays an important role in its treatment. Endocrine therapy has a limited effect, as the majority of tumors are negative for ER or PR. Thus, chemotherapy is the most important part of the treatment for lipid-rich carcinomas. In a previous study, an *in vitro* MTT assay showed that the lipid-rich tumor was more sensitive to platinum and drugs that target microtubules, such as paclitaxel and vincristine, compared with Adriamycin (3). Since HER2 overexpression is found in the majority of lipid-rich tumors, these patients may benefit from herceptin treatment (3). Radiotherapy should be administered to patients with four or more positive axillary lymph nodes according to the guidelines for the treatment of breast cancer (13).

Although only small number of cases of lipid-rich carcinoma have been reported, the prognosis of this cancer appears to be extremely poor (4,14), with two- and five-year overall survival rates of 64.6 and 33.2%, respectively (3). The median survival time is between 16 and 35 months (1,3). Previous studies have found axillary lymph node metastases in 79.2–100% of patients (1,3), and the majority of patients develop distant metastasis in the following two years (14). However, there remains no consensus on the prognostic factors with regard to lipid-rich breast carcinoma. The presence of positive axillary lymph nodes is a significant indicator for poor survival, while age, histological grade, tumor size, HER2 expression and Ki67 status remain controversial (1,5).

In conclusion, the present study analyzed the clinical features, imaging results, pathology, treatment and prognoses of two patients with lipid-rich carcinoma of the breast, which highlighted lipid-rich carcinoma as a unique type of breast cancer that should be recognized by pathologists and clinicians owing to its aggressive clinical behavior. Due to its poor prognosis, a correct diagnosis is necessary for this extremely rare neoplasm. Early diagnosis and standard oncological treatment may be of use for increasing the overall survival of patients with lipid-rich carcinoma.
